# Cognitive representations of handball tactic actions in athletes–The function of expertise and age

**DOI:** 10.1371/journal.pone.0284941

**Published:** 2023-05-04

**Authors:** Ludwig Vogel, Thomas Schack

**Affiliations:** 1 Research Group Neurocognition and Action—Biomechanics, Faculty of Psychology and Sports Science, Bielefeld University, Bielefeld, Germany; 2 Center for Cognitive Interaction Technology, Bielefeld University, Bielefeld, Germany; Mugla Sitki Kocman University: Mugla Sitki Kocman Universitesi, TURKEY

## Abstract

The tactical cooperation for the optimal interaction of team members is an essential performance-determining variable in sports games. The underlying cognitive memory structures of cooperative tactical actions have so far been little researched. Therefore, this study investigated the cognitive memory structure of tactical knowledge of handball actions in teams of different expertise and age groups. In the first experiment, tactical mental representation structures (TMRS) of 30 adult handball players of two different level of expertise were investigated. In the second experiment, TMRS of 57 youth handball players from three age levels were investigated. In both experiments the TMRS was measured with the structure dimensional analysis of mental representation (SDA-M) method. The SDA-M commences with a splitting procedure of a given set of concepts and reveals with a cluster analysis the relational structures of the concepts on an individual and a group level. Experiment one revealed that the TMRS differed significantly between skilled either/or less experienced handball players. Skilled handball players showed a hierarchical organized representation that shared more features with the basic tactical structure of the handball game than less experienced players. The second experiment revealed age-related differences of the TMRS between the age groups of the U15, U17 and U19 teams. Further data analysis revealed significant differences of the TMRS between experienced and less experienced handball players and between local and regional competition level players. We conclude that our current findings suggest that tactical expertise is mediated by elaborate cognitive tactical knowledge in memory. Furthermore, our results indicate that tactical knowledge plays a substantial role during tactical skill learning as it differs as function of age, experience, and competition level. From this point of view, team representations of game situations can be seen as a crucial factor for efficient and common interaction in fast-paced team sports.

## Introduction

The interaction of team members represents a significant performance-determining factor in competitive team sports [1, 2]. The teamwork through interaction of the team members can lead to higher as well as lower performance than the sum of the individual performances [3–5]. From a cognitive-perceptual perspective, for successful interaction, team members should have shared cognitive knowledge that enables coordinated teamwork [6]. On an individual level, tactical knowledge is the basis for goal-oriented actions and decision—making on the playing field [7]. This implies that relevant tactical information of sport situations are stored as internal representation in memory and build the cognitive basis for purposeful (inter)actions. Based on this understanding, tactical mental representation structures (TMRS) can be seen as cognitive equivalences of tactical information and situations that are stored in long-term memory. Thus, in line with the event coding theory, these cognitive structures of tactical events form the common representation of tactical knowledge, information processing and actions [8]. To investigate the cognitive structures of team sport tactics, the present study examines expertise-dependent differences in TMRS in indoor handball.

However, when considering the expertise-dependent cognitive aspects of tactical behavior, we should first consider fundamental research of expertise in sport. A wide-ranging amount of research exists on perceptual-cognitive expertise in sport, comparing performance of experts, skilled athletes, and novices [9, 10]. The results reveal distinguish attributes that systematically relate to skilled performance, e.g., that cognitive-perceptual skills seem to be domain-specific rather than general (for a review, [11]). Typically, expert performance and its development is explained in terms of skill-based theories of memory and cognitive processes derived either from psychology [12] or from ecological psychology [13]. From a cognitive-perceptual perspective, expertise yields in domain-specific adaptions with more elaborate and refined mental representations to mediate performance [14, 15]. In 1973, Chase and Simon [16] showed in chess playing that in domain-specific pattern recognition experts outperform individuals of lesser skill. Moreover, also sport experts show in their domain of expertise better recognition and recall performance than lesser skilled athletes or novices. For this, participants were asked to recall shortly presented sport specific spatial patterns. With this paradigm, research on tactical knowledge from basketball [17], field hockey [18] and indoor handball [19] indicate expertise advantages with superior performance in pattern perception, recall accuracy and decision strategies. In addition, studies of children’s tactical knowledge revealed expertise-dependent differences in tennis [14] and basketball [20]. In tennis, children with more expertise generated more tactical alternatives in game situations on verbal report measures than novices [14], also sport-specific knowledge in basketball was a significant predictor of advanced decision-making [20]. Collectively, the research on tactical knowledge helped to understand the cognitive processes and point out the crucial role of elaborate cognitive knowledge for expert performance.

Although the research of tactical knowledge provides insights into details of knowledge representation, the applied methods do not deliver detailed structure of representations for tactical decisions. However, research on cognitive structures of tactical knowledge revealed expertise-dependent differences in representational structures in soccer [21] and futsal [22]. Both studies applied the structure dimensional analysis of mental representation (SDA-M), an experimental method to investigate the structure and dimensions of in a given set of concepts. The SDA-M uses a splitting procedure to reveal psychometrically the cognitive structure and dimensions of a conceptually ordered representation [23, 24]. In comparison to other methods of measuring mental representations [25] the SDA-M provides metric data for structural knowledge analysis. The method has been already applied in research on different type of motor action [26, 27], manual actions [28, 29], workflows [30], rehabilitation [31] and intelligent systems [32]. Lex and colleagues [21] investigated mental representations and the cognitive processing of tactical situations in soccer. Therefore, they captured the long-term memory structures in an expert-novices design, and secondly investigated the gaze behavior in a tactical decision-making task. Skilled soccer players showed functionally structured TMRS compared to less skilled soccer players. In addition, the expert player reacted faster and more accurate than the novice players did. In a further study on cognitive structures of tactical representations, Frank and colleagues [22] explored in a longitudinal study paradigm the influence of tactical imagery training on mental representations. Results showed that performance improvement by imagery training during early stages of skill acquisition is accompanied by changes in the cognitive representation structure in direction of an expert representation. Based on their results, they argued that tactical skill representations change with practice over time and reasoned that functional organized representation underpin expert performance. In sum, the studies demonstrate that the TMRS in skilled athletes are characterized by well-integrated networks of shared mental representations, while novices or control groups show less structured patterns. Furthermore, both studies reveal that it is possible to assess TMRS with the SDA-M and that expertise and skill development can be assessed, respectively.

In line with these results, Nevett and French [33] and McPherson [14] suggests that young players develop shared mental representations about tactical situations and problem solving of their sport. Shared cognitions are associated with simplified decision making of teams [34], and thus more efficient and faster [19, 35]. Especially in fast-paced sport, such as indoor handball, the situation efficiency plays a critical factor for team success [36]. Thus, we investigated the TMRS of youth and adult handball players to study in how far representation structures differ in the context of age, experience, and competition level. Indoor handball is a ball game with two competing teams that are only allowed to throw the ball with the hands. Six field players and one goalkeeper per team compete on a playing court of 20 x 40 m. No field player is allowed to remain in the goal-area six meter around the goal. In ball possession, the attacking team tries to find solutions to throw the ball from a short distance or without opponent contact on the goal by applying running paths and fake actions. In comparison, the defending team tries to intercept the ball by anticipating the opponent’s actions and imposing poor scoring opportunities [37]. To investigate the TMRS with the SDA-M, we displayed game situations with images and asked handball athletes (Experiment 1) and youth handball player (Experiment 2) to conduct a specific splitting procedure (detailed description in the procedure section). The images depicted offensive, defensive, as well as transition team tactics. In the first experiment, we used a cross-sectional study design with adult athletes. We presume to replicate the findings on TMRS in soccer [21]. Therefore, we hypothesized that skilled players show more differentiated cognitive representation structures of the handball tactics compared to less skilled players. In difference to Lex and colleagues [21], the less skilled handball players had the same number of years playing handball in comparison to the skilled group. However, although the years of handball experience are similar, the current amount of training is considerably different. In the second experiment, we investigated the mental representation structure of young handball players of different age levels. According to previous research on manual action [28], we expect more elaborate TMRS in older players, no meaningful structure in young players, and expertise differences [26], that is, a functional structure organization of TMRS in more skilled players.

## Materials and methods

### Ethics statement

In both experiments, participants received no financial compensation for the participation and provided written consent prior to the experiment. Prior the experiment 2, informed consent of children’s parents was obtained. The study was approved by the Ethics Committee of Bielefeld University.

### Experiment 1

In the first experiment, mental representation structures of adult handball players of two different level of expertise were investigated with the SDA-M. Based on previous reports [21, 22] more functional and distinct tactical representation structures for skilled players were expected, since expertise is assumed to be mediated by elaborate cognitive structures [38]. Experiment 1 tested if mental representations of tactical situation differ between skill levels in handball.

### Participants

Thirty males participated in this study and were classified into two groups. The “skilled players” (n = 15, all male) were high-level handball players, playing in the third German Handball League. The skilled players mean age was 23.2 years (SD = 3.8), with a mean of 16.8 years of experience in handball and an average of 3.4 training hours per week (SD = 0.9). Three players played already in the first divisions of the German league and the SEHA-League, respectively. In addition, one player already played in the EHF-European-League. The “less skilled players” group (n = 15, all male) were experienced handball players with a mean age of 25.3 years (SD = 5.9), with an average of 1.8 training hours per week (SD = 1.1) and 17.1 years of handball experience from the 7^th^ and 8^th^ division. Prior both experiments, we determined the number of participants by a qualitative analyses of sample sizes that included invariance analysis results in the field of SDA-M studies.

### Apparatus

**Fig 1:**
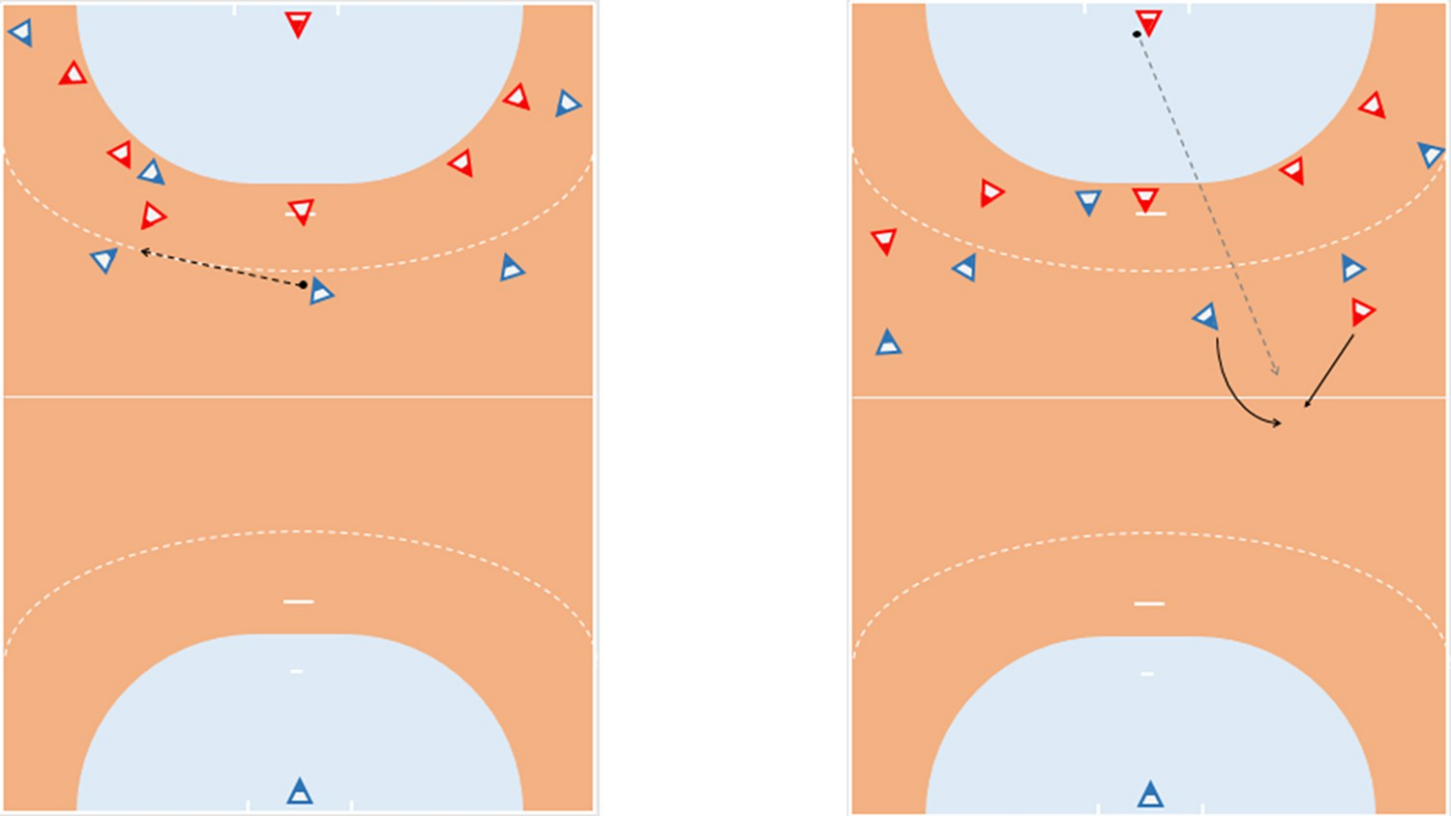
Experimental setup for the SDA-M split procedure to measure the cognitive representation. A screen showed two different tactic situations in indoor handball. The left stimulus was in an anchoring position and compared to every stimulus. Afterwards, the next randomly chosen stimulus became the anchoring stimulus until every stimulus was one time in the anchoring position. The red triangles represent the opponent team, whereas the blue triangles represent the own team. In this example, the left picture displays an offensive tactic situation of the blue team, and the right picture displays a transition from offense to defense from the blue team.

Participants of each group sat in front of a presentation, five meters away from a screen (size 2.5 x 2 m). They confirmed verbally that they could see all details on the screen. All participants had a pencil and a board with sheet to judge the presented pairs of handball situations. The stimuli consist of twelve images that were always presented as a pair. One example pair is displayed in Fig 1. All times, the participants saw the handball field with the opponent’s goal on the upper side, to facilitate the viewing perspective [39]. The displayed field included all necessary lines of the playing area and kept the conventional proportions [40].

### Stimulus

The stimuli entailed of twelve images that displayed indoor handball game situation. The design of the visual stimuli was inspired by the experiment of Lex and colleagues [7] and adapted to a handball version. Thus, the tactic situations were presented in form of a coach board that is commonly applied in team sport. From a bird’s eye perspective, the static pictures showed a handball field with all fourteen players on the pitch (Fig 1). They depicture the structural organization of a game situation and do not incorporate dynamic features or perceptual cues to avoid a perceptual resonance effect [41]. Equilateral triangles with red peaks were used to simulate the opponent position, whereas blue peaks were used to symbolize the position of the own team. The colored peaks symbolize the gaze and body orientation of the player on the field. One attacker had a black dot next to the side that symbolized the ball. Running paths were displayed with a straight line, whereas pass routes were displayed with a dotted line.

The handball game is characterized by recurring game phases that occur in a different order depending on the course of the game [42]. Within the game phases, the teams must solve the game situations successfully with technical and tactical actions individually or in cooperation with the other players. In handball, a basic distinction is made between offensive and defensive game phases. In the offensive phase of the game, the team is in possession of the ball and, through teamwork, prepares an optimal attack conclusion, e.g., a backcourt player in cooperation with the pivot creates an opportunity to throw a goal. In the defensive phase of the game, the defending team tries to disrupt the opposing team’s build-up and prevent a successful goal. To do this, the actions of the attacking team must be reacted to. If opposing players swap positions, for example, the opposing players must be handed over and taken over. In the reversal phases between the offensive and defensive game phases, it is important for the teams to initiate an attack quickly and in a well-organized manner (from defense to attack) or to successfully disrupt the opponent’s counter-initiation through a quick and effective retreat (from attack to defense). The reversal phase from defense to attack is initiated immediately after winning the ball and tries to quickly bring the ball close to the opponent’s goal. The reversal phase from attack to defense is initiated immediately after losing the ball and is intended to prevent the opponent from scoring quickly or to win the ball back directly.

In our experiment, we applied twelve stimuli consisted of the four distinctive tactic situations that are aligned to four game phases in indoor handball. In an evaluation study prior the experiment, five handball coaches (M_age_ = 41.2 years, SD = 8.4) with 17.4 years (SD = 9.3) coaching experience and trainer licenses c (n = 1), and b (n = 4) from the German Handball Association rated handball tactics (n = 28) regarding the correct and clear display of the team tactics (0 = not typical– 100 = typical). Fleiss’ Kappa [43] was calculated to assess the interrater reliability. According to Landis and Koch [44], the Kappa value denote a slight level of agreement (Κ = 0.18 < 0.2). Therefore, an Item Fit analysis [21] was conducted by the subtraction of the coefficient of variation multiplied with 100 from the mean. This incorporates the inhomogeneity of the coaches’ judgments to select the most appropriate tactic situations. From the Item Fit analysis, the three best fitting images for each tactic situations (backcourt-pivot cooperation, transition to attack, handing over–taking over, and transition to defense) were included in the experiment. The results of the Item Fit analysis are displayed in Table 1.

**Table 1:** Overview of the stimuli with the results of the SDA-M stimuli evaluation. Based on the coaches’ decisions, the item fit analysis resulted in the selection of the three team-specific tactics used per game phase. The descriptions of the team tactics refer to the participants’ team perspective.

No	Game phase	Description of the team tactic	Mean	SD	Item Fit
1	Offense	Backcourt-pivot cooperation in the center	76.00	19.49	50.35
2	Offense	Backcourt-pivot cooperation on the left side	66.00	15.17	43.02
3	Offense	Backcourt-pivot cooperation on the right side	77.00	17.18	54.69
4	Transition to attack	Steal on the right side	82.00	02.74	78.66
5	Transition to attack	Steal in the center	73.00	33.09	27.67
6	Transition to attack	Counterattack after goal throw	68.00	13.04	48.83
7	Defense	Handing over–taking over in the center	83.00	14.83	65.13
8	Defense	Handing over–taking over on the left side	81.00	31.70	41.86
9	Defense	Handing over–taking over on the right side	86.00	17.82	65.28
10	Transition to defense	Interception on the right side	89.00	11.40	76.19
11	Transition to defense	After fastbreak in the midfield	90.00	17.32	70.75
12	Transition to defense	Interception on the left side	72.00	08.37	60.38

### Procedure and data analysis

Prior to the experiment, participants were informed about the used symbols in the depicted handball situations. The participants became the instruction that they will see a pair of two images, each with a handball situation. They had to judge with + and–on their sheet whether their own team reacts in both situations similar or different. After their decision, participants were instructed to lay their pencil on the board. The next pair appeared on the screen when all participants had their pencil on the board. For the splitting procedure, the image on the left side was in anchoring position and compared to every other image (n– 1) on the right side. Every image was one time in the anchor position. Afterwards, the experimenter transferred the data into the SDA-M software QSplitV17.

The SDA-M method consists of four steps (detailed description in [45]). Initially, in the first step, participants were asked to perform a splitting procedure, that is, a pair of two stimuli was presented and participants had to judge whether their team reacts in both displayed situation in the same tactical manner (Fig 1). Each stimulus was one time in the anchor position and had to be compared with the other stimuli (n—1). Thus, twelve decision trees derived from the splitting procedure. The decisions were weighted with respect to the amount of positive and negative decisions and afterwards computed and z transformed. The second step of the SDA-M is the hierarchical cluster analysis to form individual cluster solutions that can be mapped as a dendrogram. In the third step, the Z-matrix was transferred into a correlation matrix. In the last step, the invariance analysis of the group structures revealed between group differences. Corresponding to an alpha level of p = 0.01 with a d_crit_ = 4.55, there was a significant difference between cluster solutions when λ < λ_crit_ = 0.68 [46]. Furthermore, to investigate the gradual similarity of the mean group clusters, we applied the Adjusted Rand Index (ARI) and compared each cluster solution to an ideal structure [46, 47]. The ARI ranks the similarity from -1 to +1, that is, the value +1 indicates that the cluster solutions are similar and -1 that the cluster solutions are divergent. The ideal structure was aligned to the stimuli structure and had one cluster for backcourt-pivot cooperation, one cluster for transition to attack, one cluster for handing over–taking over, and one cluster for transition to defense.

### Experiment 2

In the second experiment, TMRS of youth handball players from three age levels were investigated with the SDA-M. Based on previous reports [28] we expected more functional and distinct tactical representation structures as a function of age, experience, and competition level.

### Participants

Fifty-seven male youth handball players (M_age_ = 15.2 years, SD = 2.4) participated in this study. The players regularly competed in the youth league of their age group (U15, U17, and U19) on a local (n = 31) or regional level (n = 26). The mean age of the U15-group (n = 18) was 12.2 years (SD = 0.9), 15.6 years (SD = 0.5) for the U17-group (n = 22), and 17.8 years (SD = 0.7) for the U19-group (n = 17). The youth handball players had 1–5 years (n = 18), 6–9 years (n = 18), and ≥10 years (n = 16) handball experience and an average of 3.2 (SD = 1) training hours per week.

### Apparatus and stimulus

The apparatus and stimulus were identical to that of experiment 1 with a few exceptions. Participants in experiment 2 were tested individually on a computer with the SDA-M software QSplitV17. The decisions were given by a key press of ‘left’ and ‘right’. Furthermore, the stimuli had to be slightly adjusted to rules of youth handball games. Obligatory, youth teams are not allowed to play a defense formation that is strictly around the goal-area line. Therefore, we adjusted our stimuli by displaying the defender farer away from the goal-area line.

### Procedure and data analysis

The procedure and data analysis were identical to experiment 1.

## Results

### Experiment 1

**Fig 2:**
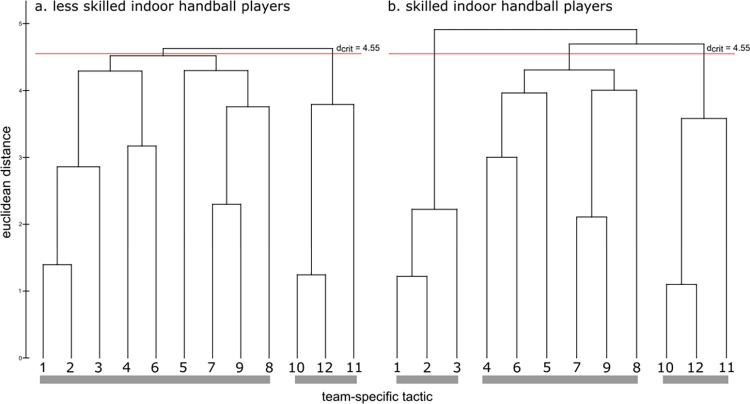
Mean group dendrograms of handball team tactics for (a) less skilled indoor handball players and (b) skilled indoor handball players. The values on the y axis display Euclidean distances and the numbers on the x axis relate to the team-specific tactic stimuli, that is, (1–3) backcourt-pivot cooperation, (4–6) transition to attack, (7–9) handing over–taking over, and (10–12) transition to defense. Connections beneath the horizontal red line are statistically considered as clusters (d_crit_ = 4.55; α = 0.01), whereas connections above this value are considered as distinct.

Representation structures for the skilled and less skilled handball players are displayed in Fig 2 as group dendrograms. The cognitive structure of the skilled group did show distinct clustering of backcourt-pivot cooperation, transition to defense, and a common clustering of transition to attack and handing over–taking over. The links of the dendrogram were below the critical value of d_crit_ = 4.55 for a given significant level of α = 0.01. In contrast, the cognitive structure of the less skilled group did show two distinct clusters with one cluster transition to defense and one cluster, with a mix from backcourt-pivot cooperation, transition to attack, and handing over–taking over. Specially, the tactic transition to attack was not represented as a cluster, the items where integrated into the clusters of backcourt-pivot cooperation (item 4 and 6) and transition to attack (item 5). Thus, skilled and less skilled handball players had different tactical representations. Invariance analysis revealed that the representation structure of the two groups were significant different λ = 0.53 < λ_crit_ = 0.68. Additionally, comparing the structures of both groups to the ideal structure, analysis indicated that skilled handball players structures shared more similar features with the ideal structure (ARI = 0.65), then less skilled player (ARI = 0.27). In sum, data of experiment one revealed that the TMRS of skilled handball players showed a hierarchical organized representation with clusters that represent team tactics in handball. In contrast, amateurs showed less organized TMRS.

### Experiment 2

**Fig 3:**
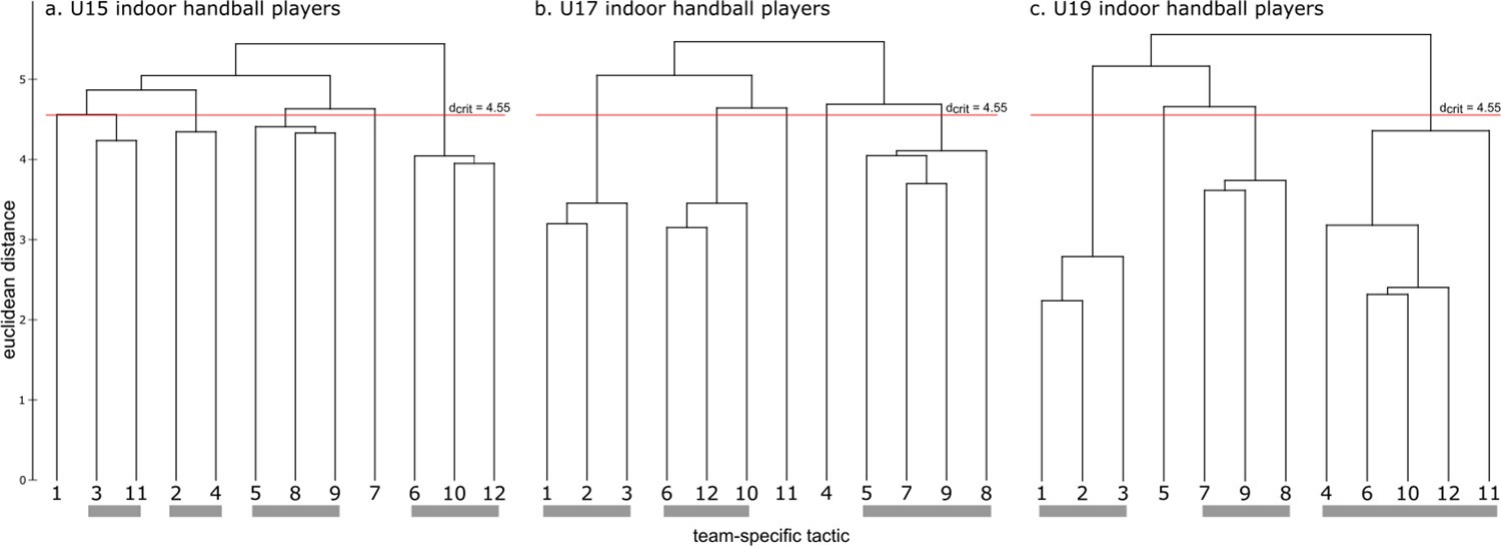
Mean group dendrograms of handball team tactics for three different age groups (a) U15, (b) U17, and (c) U19.

Representation structures for U15, U17 und U19 age groups are displayed in Fig 3 as dendrograms. The cognitive structure of the U15 did not show any distinct clustering of handball team tactics. The links of the dendrogram were all above the critical value of d_crit_ = 4.55 for a given significant level of α = 0.01. In contrast, the cognitive structure of the U17 and U19 showed distinct clustering of team tactics. The dendrograms of the U17 showed three distinctive clusters. However, two items (4 and 11) can be considered as singles, as the links to the other items were above the critical value. The cognitive structure of the U19 showed three distinctive clusters and one single (item 5), two clusters were identical to the clusters of the ideal solution. The invariance analysis revealed significant differences between the cluster solutions of the three age groups (λ < λ_crit_ = 0.68), U15—U17 λ = 0.42, U15—U19 λ = 0.43, U17—U19 λ = 0.54. Furthermore, the ARI revealed more similarity between the U19 dendrogram and the ideal structure (ARI = 0.64), then the U17 (ARI = 0.49), and the U15 (ARI = 0.06) (Table 2).

**Table 2:** Comparison of mean group dendrograms for age group, handball experience, and competition level (p = 0.01) with the ideal structure applying the ARI.

Group	ARI to ideal structure
**Current age group**	
U15 (n = 18)	0.06
U17 (n = 22)	0.49
U19 (n = 17)	0.64
**Handball experience in years**	
1–5 (n = 18)	0.61
6–9 (n = 18)	0.67
≥10 (n = 16)	0.75
**Competition level**	
Local level (n = 31)	0.39
Regional level (n = 26)	0.58

**Fig 4:**
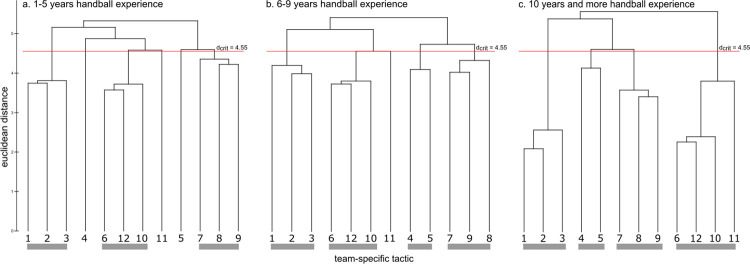
Mean group dendrograms of handball team tactics for three different groups of handball experience (a) 1–5 years, (b) 6–9 years, and (c) ≥ 10 years.

Representation structures for the three experience levels (1–5, 6–9, and ≥10 years) are displayed in Fig 4 as dendrograms. The cognitive structure of the 1–5 years’ experience group showed three distinct clusters and three singles of handball team tactics. Two clusters were identical to the ideal structure clusters. In contrast, the cognitive structure of the 6–9 and the ≥10 years’ experience group showed both four distinct clusters of team tactics. Two of the four clusters were similar to the clusters of the ideal structure, and the remaining clusters shared similar features. The invariance analysis showed significant differences between the 1–5 and both the 6–9 (λ = 0.58) and the ≥10 (λ = 0.49) years’ experience group (λ < λ_crit_ = 0.68). No differences were found between the 6–9 and the ≥10 years’ experience group (λ = 0.71). In addition, the ARI revealed more similarity between both the 6–9 years’ (ARI = 0.67) and the ≥10 years’ experience group (ARI = 0.75) to the ideal structure, then the 1–5 (ARI = 0.61) years’ experience group (Table 2).

**Fig 5:**
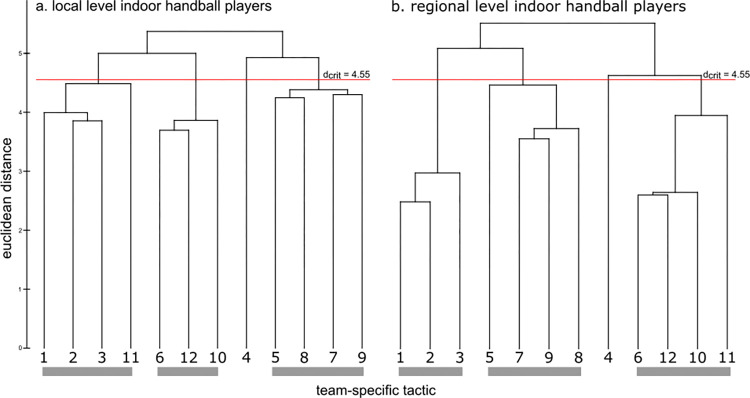
Mean group dendrograms of handball team tactics for (a) local level indoor handball players and (b) regional level indoor handball players.

Representation structures for competition levels (local and regional) are displayed in Fig 5 as dendrograms. The cognitive structure of the local level group did not show any distinct clustering of handball team tactics. The links of the dendrogram were all above the critical value of d_crit_ = 4.55 for a given significant level of α = 0.01. In contrast, the cognitive structure of the regional group showed three distinct clusters and one single (item 4) of team tactics. The invariance analysis revealed significant differences between the cluster solutions (λ = 0.58 < λ_crit_ = 0.68). Furthermore, the ARI revealed more similarity between the regional group dendrogram (ARI = 0.58) compared to the local group dendrogram (ARI = 0.39) (Table 2). Summarized, the results of experiment two showed that the TMRS of young handball players differed as a function of age, experience, and competition level. The higher the competitive level was and the older and more experienced the players were, the more similar the TMRS were to the ideal structure.

## General discussion

The present study revealed in experiment 1 differences in the TMRS of skilled and less skilled adult handball players. In experiment 2, youth handball players showed age-, experience-, and expertise-depended differences in the TMRS. Research of TMRS in soccer revealed differences of skilled and less skilled adult players as well, but it was unclear whether expertise or experience made the differences. Thus, the results of this study are consistent with earlier tactical representation research in soccer [21]. In comparison to Lex and colleagues [21], the present study examined less-skilled handball players with more domain-specific experience than the soccer novices (17.1 years of handball experience vs. 3.2 years of soccer experience). However, the experience in years between the skilled and the less-skilled players in this experiment was nearly similar, the current cumulated hours of practice differ due to the almost double amount of practice per week of the skilled players. Even though the current training times differ, we cannot preclude the possibility that the training histories in the youth and/or adult divisions were the same or similar. Nevertheless, as predicted, we found significant differences of the cognitive tactic structures between skilled and less skilled players. Skilled players represented offensive and transition team tactics next to the opponent’s goal as separate units, whereas team tactics in the own half field were unexpectedly not separated. From a functional perspective, this interesting result could be explained with anticipatory defense strategies that share the common goal to intercept the ball. Thus, both team tactics transition to attack and handing over–taking over are functional related and consequently represented as one unit in the team cognition. The analysis of the cognitive structure of the less-skilled players also showed no separate unit for the team tactic transition to offense. Partly, the tactics were integrated in the offensive clusters of backcourt-pivot cooperation. The separation in offensive and defensive team tactics is similar to patterns found in novice soccer players [21]. The findings suggest that novices and less skilled player represent the team tactic related to offensive and defensive tactics, whereas skilled players represent defense and transition to offense tactics together, that is, skilled players represent team tactics goal-oriented to conquer the ball. However, further research is warranted to examine this assumption.

As predicted, we found no functional cognitive structures of team tactics in young (U15) handball players. Consistent with predictions, youth U17 and U19 players’ representation structures were increasingly more similar to the ideal structure. Further, we found differences in the cognitive structure between handball beginners (1–5 years of experience) and intermediate experienced players (6–9 years), but no difference between the intermediate and the experience players (≥10 years). Moreover, youth handball players that played at a local level showed no meaningful clustering. As predicated, regional playing youth handball players showed a more functional TMRS that shared more features with the ideal structure, compared the local playing handball players. The current findings suggest that age, higher competition level and, especially in the beginner phase, more years of practice, play a crucial role in for sophisticated TMRS. However, the invariance of the cognitive structures from intermediate to high experience could be a hint that experience forms the cognitive representation in the beginning of tactical learning but should also be aligned with meaningful (tactical) experience [7]. Thus, expertise and elaborate representation cannot be explained by the sum of all hours of practice [11], but rather as a consequence of the quality and quantity of training practice [48].

Furthermore, the comparison between the cognitive structure of youth (Experiment 2) and adults (Experiment 1) revealed that youth handball players do not possess as elaborate TMRS as adult skilled, and less skilled players. This result is in line with findings from tennis [14] and baseball [33]. It is assumed that the development of tactical knowledge requires specific type of practice and takes longer than normally presumed. Accordingly, future research should identify practices and methods that help to build functional representations [49].

Although more research is warranted, our findings indicate that expertise TMRS are well-integrated functional networks that reflect the functional structure of the team tactics. Especially, the team tactic transition to offense was not well integrated in less skilled and youth players. Thus, from an applied perspective, special training methods can be used to force elaborate TMRS. For instance, handball teams should not practice defense drills individually, instead the transition to offense should be integrated as often as possible. Thus, the goal of the defense actions would shift from stopping the opponent to intercepting the ball.

One limitation of our study is that the current findings are reduced to the selected team tactics. Thus, to investigate sport overarching effects, future research could integrate more basic tactics that are applicable in various sports. First, this would provide evidence to advance theory about the cognitive structure of team tactics. Second, the influence of cognitive tactical structure on sport overarching transfer effects could be investigated [50]. Thus, future research should bridge the gap between cognitive representation and performance. This may indicate that tactical knowledge in long-term memory might be a basis for appropriate joint actions in handball. Further studies could take the performance and actual decisions into account. Additionally, the individual capabilities and the individual interpretation of tactics could lead to different TMRS. Thus, individual representations can be analyzed to detect problematic tactical structures or to help coaches to support their athletes more individually in the training process and in the game. Consequently, the measurement of TMRS in handball can be used as an assistive diagnostic tool.

In conclusion, the present study gave insides in the cognitive representation of handball team tactics in both athletes and youth players. Results suggest that expertise is mediated by elaborate cognitive structures of handball tactics. Furthermore, current findings indicate that cognitive tactical structures differ as a function of age, experience and competition level. We assume that building individual and team representations of game situations are crucial factors for efficient and common interaction in fast-paced team sports. Therefore, identifying practices and methods that stimulate higher order processing in team cognitions could be a sophisticated approach to understand the nature of tactical representation and sport expertise.

## Supporting information

S1 File Information about the participants of experiment 1 and 2. (XLSX)

S2 File Data from experiment 1. (XML)

S3 File Data from experiment 2. (XML)
